# Health and function of participants in the Long Life Family Study: A comparison with other cohorts

**DOI:** 10.18632/aging.100242

**Published:** 2011-01-10

**Authors:** Anne B. Newman, Nancy W. Glynn, Christopher A. Taylor, Paola Sebastiani, Thomas T. Perls, Richard Mayeux, Kaare Christensen, Joseph M. Zmuda, Sandra Barral, Joseph H. Lee, Eleanor M. Simonsick, Jeremy D. Walston, Anatoli I. Yashin, Evan Hadley

**Affiliations:** ^1^ University of Pittsburgh, Graduate School of Public Health, Department of Epidemiology, Center for Aging and Population Health, Pittsburgh, PA 15213,USA; ^2^ University of Pittsburgh, School of Medicine, Division of Geriatric Medicine, Pittsburgh, PA 15261, USA; ^3^ Department of Biostatistics, Boston University School of Public Health, Boston, MA 02118, USA; ^4^ Division of Geriatrics, Department of Medicine, Boston University Medical Center, Boston, MA 02118, USA; ^5^ The Gertrude H. Sergievsky Center and the Taub Institute for Research on Alzheimer's Disease and the Aging Brain, Columbia University, New York, NY 10032, USA; ^6^ The Danish Aging Research Centre, Epidemiology, University of Southern Denmark, Odense, Denmark; ^7^ National Institutes of Health, National Institute on Aging, Bethesda, MD 20892, USA; ^8^ Johns Hopkins Medical Institutions, Bayview Medical Center, Baltimore, MD 21224, USA; ^9^ Duke University, Center for Demographic Studies, Durham, NC 27708

**Keywords:** longevity, exceptional survival, family studies, genetics, healthy aging, genome wide association study, multicenter studies, aging phenotypes

## Abstract

Individuals from families recruited for the Long Life Family Study (LLFS) (n= 4559) were examined and compared to individuals from other cohorts to determine whether the recruitment targeting longevity resulted in a cohort of individuals with better health and function. Other cohorts with similar data included the Cardiovascular Health Study, the Framingham Heart Study, and the New England Centenarian Study. Diabetes, chronic pulmonary disease and peripheral artery disease tended to be less common in LLFS probands and offspring compared to similar aged persons in the other cohorts. Pulse pressure and triglycerides were lower, high density lipids were higher, and a perceptual speed task and gait speed were better in LLFS. Age-specific comparisons showed differences that would be consistent with a higher peak, later onset of decline or slower rate of change across age in LLFS participants. These findings suggest several priority phenotypes for inclusion in future genetic analysis to identify loci contributing to exceptional survival.

## INTRODUCTION

Longevity and relative longevity with preserved physiologic function or low levels of disease are types of “exceptional survival,” a term promoted by the National Institute on Aging Advisory Panel on Exceptional Longevity [1] to encompass multiple outcomes for the study of healthy aging. The term “exceptional survival” refers to “important survival outcomes such as survival without disease or disability, as well as to longevity per se.” Compression of disability and/or morbidity towards the end of very long lives has been noted to be associated with exceptional longevity which in turn has been found to be strongly familial [2-4].

The Long Life Family Study (LLFS) is a multi-center effort to enroll families clustered for exceptional survival in order to identify environmental and genetic factors that promote long healthy lives in these family members. The recruitment of families into the LLFS focused on selecting families with multiple exceptionally old living individuals. The offspring generation has not yet had the opportunity to manifest longevity; this will take years of follow-up. However, their exceptionality might manifest as lower prevalence of diseases that are the major contributors to mortality in older adults as has been demonstrated in the offspring of centenarians and the Framingham Heart Study (FHS) [5,6]. Because of more robust health or absence of disabling disease, they may also show unusually high physical and cognitive function for their age. The initial examination of LLFS participants was designed to capture several of these exceptional survival phenotypes.

This paper compares the prevalence of disease as well as physical and cognitive functioning in LLFS probands and offspring to two community based cohorts that were not selected for longevity. These comparison cohorts include the Cardiovascular Health Study (CHS), the FHS original and FHS offspring cohorts. We also compared LLFS participants to the New England Centenarian Study (NECS) [7] proband generation with the expectation that LLFS may show a similar or somewhat lower degree of exceptionality compared to this long lived cohort.

## METHODS

### Long Life Family Study

The Long Life Family Study is a family-based cohort study designed to determine both genetic and behavioral/environmental risk factors for familial exceptional survival traits, enrolling 4559 long-lived probands and their siblings (n=1445), their offspring (n=2329) and spouse controls (n=785). Family members (n=119) with only telephone interview data and a blood sample were excluded from these analyses. Thus, 3655 LLFS individuals with familial longevity (probands, siblings and their children) were compared to the other cohorts and the 785 spouse controls. Telephone follow-up is ongoing to ascertain changes in health, function and vital status.

### Recruitment

The U.S. field centers used Center for Medicare and Medicaid Services lists of Medicare enrollees to mail a recruitment brochure. The initial file included people who were at least 79 years old on January 1, 2005; had no recorded date of death; were not in the end-stage renal disease or hospice programs; lived in zip codes “near” (within 3 hours driving distance) one of the 3 U.S. study centers (Boston University Medical Center in Boston MA, Columbia College of Physicians and Surgeons in New York City NY, and the University of Pittsburgh in Pittsburgh PA). A pilot mailing tested the yield of families recruited from mailing to individuals in their 80's and higher age strata. Based on these yields, subsequent mailings targeted those age 89 and older. Study participants were also recruited from local communities using mailed brochures, posters, web-based media and newspaper advertisements as well as community-based presentations. Additional mailing lists were obtained through voter registries and purchased public domain lists from various commercial vendors.

The University of Southern Denmark field center identified individuals who would be ages 90 and above during the study recruitment period through the Danish National Register of Persons, which contains current information on names, including past names such as maiden names for women, addresses, place of birth, marriages, and vital status [8]. Second, using information on the place of birth and the names, parish registers available in regional archives were searched to locate the parents of the elderly individuals in order to identify sibships. Based on the above information, potentially eligible families were identified and contact was made with potential probands to further assess the family's eligibility for and willingness to participate in the LLFS using criteria parallel to that used in the United States.

### Eligibility and Enrollment

All potential probands were pre-screened for eligibility by telephone. Potential probands were asked questions about their family, including birth and/or death dates of all their brothers and sisters. The Family Longevity Selection Score (FLoSS) [9] was calculated to rank sibships by current age or age at death of siblings, the size of the sibship and the number of alive individuals available for study. This ranking led to the enrollment of families with the greatest potential utility for phenotypic and genetic studies of exceptional survival in families.

If a proband's family was FLoSS eligible, defined as a score of 7 or higher, they also had to meet the following criteria: 1) the proband, at least one living sibling, and one of their living offspring (minimum family size of 3) were all able to give informed consent, and 2) were willing to participate in the interview and examination including the blood sample for serum and DNA extraction. The probands were asked to contact other potentially interested family members for enrollment. Prior to examination, written informed consent was obtained from all enrollees. In a few cases of cognitive impairment, family members were enrolled via proxy consent, provided that the participant was able to express assent at the time of the examination.

### Examination

The interviews and examinations were conducted in the home setting with portable equipment by centrally trained and certified research assistants using a common protocol. U.S. research technicians also traveled to examine families and family members outside of the field center regions (about 20% of the U.S. sample) when the family was highly exceptional (FLoSS ≥15) or to enroll additional family members who resided outside of the field center regions. If an in-person visit was not feasible, a comprehensive telephone interview was conducted and a blood or saliva sample was obtained by an outside laboratory or physician's office.

Date of birth was validated using a driver's license, birth certificate or other official document or source. Sex, race, ethnicity, and education were self-reported. History of disease was assessed by self-report of a physician diagnosis and approximate date of onset for the following: heart disease (heart attack or myocardial infarction, and/or coronary artery bypass surgery), stroke (stroke and/or transient ischemia attack), hypertension, diabetes, chronic lung disease (emphysema, chronic bronchitis and/or asthma), peripheral artery disease and cancer.

Standing height was measured using a Handi-stat set square (Perspective Enterprises, Portage, MI) to the nearest 0.1 cm. Weight was measured using an electronic digital scale (SECA 841, Hanover, MD) to the nearest 0.1 kg. Body mass index (BMI) was calculated as weight (kg)/height (m)^2^. Sitting blood pressure was measured using an automated blood pressure machine (BP-tru BPM 300, VMS MedTech, Coquitlam, Canada), averaging three measures. Pulse pressure was calculated as systolic blood pressure (SBP) minus diastolic blood pressure (DBP). Lung function was measured with a portable spirometer (EasyOne™, ndd Medical Technologies, Andover, MA) using American Thoracic Society guidelines. Ankle-arm index, the ratio of the ankle to arm systolic blood pressure, was used to assess peripheral arterial disease. Physical function was assessed with questions regarding difficulty with activities of daily living (ADL's), instrumental activities of daily living (IADL's) and mobility. Performance measures of function included gait speed, chair stands and standing balance [10]. Grip strength (average of two trials) was measured using an isometric dynamometer (Jamar Hydraulic Hand Dynamometer, Lafayette, IN) in a seated position to the nearest 2 kg. Cognitive testing included the Mini-Mental State Exam (MMSE) [11] and the Digit Symbol Substitution Test (DSST) [12]. Total cholesterol, high density lipoprotein – cholesterol (HDL-C), low density lipoprotein – cholesterol (LDL-C), triglycerides, creatinine and blood glucose were assessed after at least a 6 hour fast and analyzed by the LLFS central laboratory based at the University of Minnesota.

Reported hypertension and diabetes were confirmed by specific medication use based on a medication inventory of all prescription and over-the-counter medication taken in the past two weeks. Medications were coded into major categories, including anti-hypertensives, anti-anginals, oral hypoglycemics and insulin, lipid lowering drugs and “other”. For analysis, hypertension was defined as SBP ≥ 140 mmHg or DBP ≥ 90 mmHg or self-report confirmed by use of anti-hypertensive medications. Diabetes was defined as use of diabetes medications or fasting glucose ≥ 126 mg/dL. Three ADLs were assessed in LLFS comparably to other studies: bathing/showering, walking up 10 steps and getting in/out of a bed or chair. ADL difficulty was defined as having difficulty with at least one of these three items. Detailed cognitive testing and personality [13] were assessed and are reported separately. Other behavioral and environmental characteristics assessed included past and current physical activity levels [14] and smoking history, defined as current, past, or never.

### Comparison Cohorts

The Framingham Heart Study and the CHS included many of the same measures as were used in LLFS. The Cardiovascular Health Study is an ongoing prospective, observational study designed to determine the risk factors for, and consequences of, cardiovascular disease in older adults. A total of 5,888 participants across four U.S. field centers were recruited [15]. Data from the baseline examination, conducted in 1989-1990 (and 1992-1993 for the 687 minorities) were used for the comparison. The Framingham Heart Study original cohort consisted of 5,209 respondents in 1948 from a random sample of adult households in Framingham, Massachusetts, age 30 to 62 years. Because specific exam components were comparable with LLFS, data from exam 26 (1998-2001) were used for the comparison.[16] The Framingham Heart Study offspring cohort was initiated in 1971 [17]. A sample of 5,124 men and women, consisting of the offspring of the FHS original cohort, their spouses and random sample of the Framingham population was included. Data from the FHS offspring cohort exam [7] (1998-2001) were used for this comparative analysis. The New England Centenarian Study began in 1994 as a population-based study of all centenarians living within 8 towns in the Boston area [18]. The New England Centenarian Study has gone on to enroll subjects age 96 years old and older (average age 103 years), their siblings and offspring from throughout the United States.

For each cohort, items were selected that were assessed in a comparable fashion to LLFS [15,18,19]. In the CHS and NECS, self-reported medical history items were similar to LLFS. For the Framingham cohorts, heart disease, stroke and cancer were adjudicated by review of records. For heart disease, components of the diagnosis were not available to make it comparable with LLFS, and therefore, it was not used in this analysis. Hypertension and diabetes definitions were constructed to be similar across studies, combining self-report, measured blood pressure or blood glucose and medication use. Three self-reported disability items were comparable between LLFS and CHS, but differed for NECS and FHS. The protocols for the MMSE were the similar across studies [15,18,19] and DSST was similar between CHS and LLFS [15]. Performance measures of gait speed and grip strength were assessed in CHS and FHS with slightly different protocols, but all reported results in meters per second. In CHS, the course was 15 feet compared to 4 meters in both Framingham cohorts and LLFS. Grip strength was assessed with the forearm supported in CHS and FHS and not supported in LLFS, making it somewhat more challenging. Anthropometry protocols, smoking status, and medication use definitions were comparable. Blood was drawn in the fasting state, except in the FHS original cohort, where a random sample was obtained, thus fasting glucose was not available in that cohort. All assays were conducted using standard protocols for each study in a central lab [15,19].

### Statistical Analysis

Descriptive means and proportions were assessed for LLFS overall as well as for each generation. For dichotomous outcomes, the LLFS cohort was stratified by generation, and adjusted pairwise comparisons were performed with the age range of each cohort restricted to match the age range of the LLFS generation to which it was compared. For example, the oldest (proband) LLFS generation had members age 72 to 100 years. In this generation's comparison with CHS participants, the analysis group was limited to an age range of 72 to 100 years in CHS. Similarly, the analysis comparing the offspring LLFS generation to CHS was limited to participants aged 65 to 88 years, with this range including members from both CHS and LLFS offspring generation cohorts. Thus, each comparison shows a different subset of LLFS. We were not able to match for birth cohort. For continuous variables, similar generation-stratified analyses were performed using generalized linear models adjusted for age, race, sex, education level, and smoking status with means and frequencies centered at the LLFS cohort means for the generation in the analysis. Additionally, clustering of familial traits in LLFS and FHS was adjusted for by controlling for family membership using an exchangeable correlation structure. Models examining creatinine were additionally adjusted for height and weight. Blood pressure means were adjusted for anti-hypertensive medication use and lipid levels were adjusted for use of lipid lowering medications. Interactions for age and sex were tested in all models and none were statistically significant. SAS version 9.1 (Cary, NC) and the GEE package in R version 2.7 were used to perform the analyses.

## RESULTS

The Long Life Family Study cohort had a wider range of ages than any of the other cohorts (Table 1). The mean age in CHS was similar to LLFS, though limited to age 65 and over at the younger end of the distribution. The Framingham Heart Study original cohort was older than both LLFS and CHS, while the FHS offspring cohort was more similar in age to LLFS. The NECS had the oldest individuals, with a mean age of 102.8 years, ranging from 90 to 119 years, similar to the LLFS probands generation. The Long Life Family Study cohort was more highly educated compared to CHS, NECS and FHS and this was more apparent in the LLFS offspring generation. History of smoking was similar among LLFS, CHS and FHS, with about 40-50% of participants with no history of smoking. In the NECS, 80% of participants reported that they never smoked.

**Table 1.** Socio-demographic, health and functional characteristics of LLFS and comparison cohorts.

	LLFS*	LLFS Probands*	LLFS Offspring*	CHS	FHS-Original	FHS-Offspring	NECS	LLFS Controls
	**N = 3690**	**N = 1373**	**N = 2317**	**N = 5888**	**N = 542**	**N = 3475**	**N = 1088**	**N = 582**
***Socio-Demographic***								
**Age**								
Mean± SD	72.5± 16.4	91.0 + 6.1	61.0 + 8.3	72.8± 5.6	86.2± 4.1	61.4± 9.6	102.8± 4.04	61.8± 8.6
Range	31 - 110	72 - 110	31 - 88	65 - 100	79 - 103	33 - 90	90 - 119	25 - 88
**Gender (%)**								
Male	44.1	47.0	42.3	42.4	30.3	46.0	27.4	52.1
Female	55.9	53.0	57.7	57.6	69.7	54.0	72.6	47.9
**Race (%)**																
White	99.3	98.9	99.6	83.6	100.0	100.0	97.4	99.0
Non-White	0.7	1.1	0.4	16.4	0.0	0.0	2.6	1.0
**Education (%)**																
Less than High School	13.8	26.4	6.0	29.5	25.4	5.8	60.6	11.0
High School or equivalency	13.4	22.1	8.1	27.6	39.0	33.1	18.7	4.5
More than High School	72.8	51.5	85.9	42.9	35.6	61.1	20.8	84.5
**Smoking Status (%)**																
Never Smoked	54.4	57.6	52.3	46.6	39.0	36.2	80.2	49.8
Former Smoker	38.3	39.5	37.6	41.5	57.0	50.0	17.3	41.0
Current Smoker	7.3	2.9	10.1	11.9	4.0	13.8	2.5	9.2
***Medication Use* (%)**								
Lipid-Lowering Medications	16	19	14	5	23	21		16
Anti-Hypertensive Medications	43	67	28	31	62	34		28
***Disease Prevalence* (%)**								
Heart Disease	9.2	17.7	3.9	11.9	N/A	N/A	10.0	5.7
Stroke	7.7	15.9	2.5	4.3	10	3.0	15.7	3.6
Hypertension	52.2	66.3	43.4	54.5	82.6	37.1	37.6	46.6
Diabetes	4.7	6.0	3.9	8.5	36.6	11.7	6.7	3.4
Cancer	15.5	23.5	10.5	14.3	22.7	8.8	21.1	12.7
Chronic Obstructive Lung Disease	6.7	8.2	5.7	23.8	N/A	N/A	4.4	13.1
Peripheral Artery Disease	7.0	17.9	0.9	13.4	N/A	2.6	9.3	1.7
Activities of Daily Living, difficulty with one or more	18.0	40.1	4.4	14.3	N/A	N/A	N/A	7.6
***Physical Measures* (Mean± SD)**								
Height (cm)	165.1± 10.7	160.1 + 10.3	168.0 + 9.7	164.8± 9.5	159.9± 9.2	167.5± 9.4		169.9± 9.2
Weight (kg)	74.2± 17.2	66.9 + 14.0	78.5 + 17.5	72.7± 14.7	66.2± 13.5	79.1± 16.8		79.3± 16.0
Body Mass Index (kg/m^2^)	27.1± 5.2	26.0 + 4.3	27.8 + 5.5	26.7± 4.7	23.3± 4.3	28.2± 5.3		27.4
Gait Speed (m/s)	1.00± 0.32	0.72 + 0.26	1.17 + 0.22	0.86± 0.22	0.80± 0.22	N/A		1.2
Cholesterol (mg/dL)	198.7± 42.3	187.5 + 43.5	205.6 + 40.1	211.2± 39.3	185.4± 36.2	200.3± 36.8		207.1± 41.0
High-Density Lipoprotein Cholesterol (mg/dL)	58.6± 17.4	55.9 + 16.0	60.3 + 18.0	54.2± 15.7	55.0± 17.5	53.7± 17.0		58.7± 16.7
Low-Density Lipoprotein Cholesterol (mg/dL)	117.6± 35.5	109.4 + 35.6	122.6 + 34.5	129.8± 35.7	101.0± 31.4	119.4± 33.2		125.9± 34.9
Triglycerides (mg/dL)	113.4 + 70.8	110.4 + 59.8	115.2 + 76.8	139.6± 76.9	146.8± 75.6	137.1± 89.0		116.7± 83.8
Creatinine (mg/dL)	1.06 + 0.35	1.20 + 0.44	0.98 + 0.26	1.07± 0.4	1.24± 0.42	1.07± 0.32		0.99± 0.20
Forced Vital Capacity (L)	3.07 + 1.04	2.26 + 0.77	3.49 + 0.91	2.96± 0.87	N/A	N/A		4.47± 14.9
Ankle-Arm Index	1.16 + 0.18	1.07 + 0.21	1.21 + 0.13	1.06± 0.18	N/A	1.15± 0.12		1.21± 0.14

* Values are provided for entire cohorts, subsequent comparisons restricted to match age ranges of probands and offspring.

Table 2a presents the odds ratios for disease prevalence in the LLFS proband generation cohort compared to similarly aged subsets of the other comparison cohorts. The odds ratio for heart disease, stroke and hypertension was similar to or lower in the comparison cohorts compared to LLFS probands except for the FHS original cohort which had had a significantly higher risk of hypertension compared to the LLFS cohort. In the case of diabetes, FHS offspring cohort had higher prevalence compared to LLFS probands; there was no difference in diabetes between CHS or NECS participants. The prevalence of cancer was significantly lower for FHS offspring cohort compared to the LLFS cohort, with no differences found between the other cohorts and LLFS. Self-reported history of lung disease was more than three times more prevalent in the CHS cohort, compared to the LLFS, p<0.0001. The risk of having peripheral artery disease (defined as an ankle-arm index <0.9) was three times more likely in the CHS compared with the LLFS participants. Further, the risk of gait speeds of <1.0 m/s were nearly four times more likely for the CHS participants as compared to LLFS, p<0.0001.

**Table 2a.** LLFS Probands: Odds ratios for disease and disability prevalence in comparison cohorts relative to LLFS; adjusted for age, sex, race, education and smoking status.

	LLFS	CHS (N=2967)		FHS -Original (N=519)		FHS-Offspring (N=541)		NECS-Proband (N=1088)	
	OR	OR	(95% CI)	OR	(95% CI)	OR	(95%CI)	OR	(95%CI)
Heart Disease	1.00	0.68	(0.51–0.91)**		N/A		N/A	0.89	(0.57–1.41)
Stroke	1.00	0.52	(0.35–0.76)***	0.69	(0.47–0.99)*	1.02	(0.72–1.45)	0.88	(0.55–1.34)
Hypertension	1.00	0.96	(0.76–1.20)	1.84	(1.36–2.49)***	0.57	(0.50–0.65)**	0.48	(0.34–0.67)***
Diabetes	1.00	0.82	(0.56–1.22)		N/A	2.89	(2.17–3.84)**	1.93	(0.97–3.84)
Cancer	1.00	0.79	(0.61–1.03)	1.02	(0.77–1.34)	0.71	(0.58–0.87)**	1.00	(0.68–1.47)
Chronic Obstructive Lung Disease	1.00	3.40	(2.53–4.56)***		N/A		N/A	0.56	(0.30–1.07)
Peripheral Artery Disease	1.00	3.00	(2.15–4.19)***		N/A	1.70	(0.95–3.05)		N/A
Activities of Daily Living, difficulty with one or more	1.00	1.28	(0.97–1.69)		N/A		N/A		N/A
Gait Speed, <1.0 m/s	1.00	3.99	(2.95–5.40)***	1.14	(0.80–1.63)		N/A		N/A

LLFS N's: CHS vs. LLFS N = 1382; FHS-Original vs. LLFS N = 1366; FHS-Offspring N = 573; NECS-Proband vs. LLFS N = 954

* p ≤ 0.05

** p ≤ 0.01

*** p ≤ 0.001

Table 2b presents the odds ratios for disease prevalence in the LLFS offspring generation cohort and in the similarly aged subsets of CHS, FHS original, FHS offspring, and LLFS spousal controls. Heart disease prevalence among CHS participants was more than two times higher compared to the LLFS offspring. There were no differences in self-reported history of stroke among the CHS, FHS original, FHS offspring and the LLFS cohort. However, the odds of stroke were more than two-fold higher for LLFS spouse controls compared to LLFS offspring. CHS and FHS original cohorts had significantly lower risk of hypertension compared to the LLFS offspring. For diabetes, CHS and FHS offspring cohorts had higher prevalence compared to LLFS offspring. The prevalence of cancer was significantly lower by 20% for the FHS offspring cohort compared to the LLFS cohort, with no differences found between the other cohorts and LLFS offspring. The CHS cohort had a nearly 2.5-fold greater prevalence of self-reported history of lung disease compared to the LLFS. Peripheral artery disease risk was three times more likely in the CHS compared with the LLFS offspring cohort. In addition, the risk of gait speeds of ≤1.0 m/s were nearly five times more likely for the CHS participants as compared to LLFS offspring, p<0.0001.

**Table 2b.** LLFS Offspring: Odds ratios for disease and disability prevalence in comparison cohorts relative to LLFS, adjusted for age, sex, race, education and smoking status.

	LLFS	CHS (N=5798)		FHS-Original (N=367)		FHS-Offspring (N=2946)		LLFS Controls (N=777)	
	OR	OR	(95% CI)	OR	(95% CI)	OR	(95%CI)	OR	(95%CI)
Heart Disease	1.00	2.12	(1.72–2.63)***		N/A		N/A	0.93	(0.42–2.08)
Stroke	1.00	0.81	(0.63–1.04)	0.69	(0.46–1.03)	1.04	(0.73–1.49)	2.31	(1.18–4.51)*
Hypertension	1.00	0.86	(0.74–0.99)*	1.97	(1.44–2.68)***	0.74	(0.64–0.85)***	1.12	(0.89–1.40)
Diabetes	1.00	1.45	(1.06–2.01)*		N/A	5.41	(3.77–7.77)***	0.89	(0.42–1.87)
Cancer	1.00	0.97	(0.80–1.18)	0.99	(0.76–1.32)	0.79	(0.64–0.98)*	0.97	(0.63–1.50)
Chronic Obstructive Lung Disease	1.00	2.44	(1.99–2.99)***		N/A		N/A	1.19	(0.76–1.88)
Peripheral Artery Disease	1.00	2.97	(2.19–4.03)***		N/A	1.39	(0.85–2.26)	2.93	(0.95–9.02)
Activities of Daily Living, difficulty with one or more	1.00	1.03	(0.84–1.26)		N/A		N/A	1.09	(0.66–1.81)
Gait Speed, <1.0 m/s	1.00	4.76	(3.92–5.80)***	1.32	(0.91–1.92)		N/A	0.77	(0.57–1.06)

LLFS N's: CHS vs. LLFS N =771; FHS-Original vs. LLFS N = 50; FHS-Offspring N = 2312; LLFS Controls vs. LLFS N = 2732

* p ≤ 0.05

** p ≤ 0.01

*** p ≤ 0.001

Adjusted mean differences in blood chemistries and physical characteristics were compared for LLFS, CHS, and FHS cohorts (Table 3a and 3b). LLFS proband generation participants had higher BMI compared to all other cohorts, but this was only statistically significant compared to the CHS cohort. For the LLFS offspring generation, BMI was higher compared to both the CHS and FHS original cohorts. Mean grip strength was higher for LLFS probands compared to FHS original cohort however, it was lower compared to CHS. There were no statistically significant differences in grip strength between the LLFS offspring cohort and any of the comparison groups. Long Life Family Study participants in both the proband and offspring generation had significantly faster walk speeds compared to CHS. Similarly, digit symbol substitution test scores were significantly better for both LLFS cohorts compared to CHS. Additionally, there was a slight difference between the LLFS proband generation and FHS offspring cohort for the MMSE.

**Table 3a.** LLFS Probands: Measurement means in LLFS and comparison cohorts r adjusted for age, sex, race, education and smoking status.

	Cardiovascular Health Study			Framingham Heart Study – Original Cohort			Framingham Heart Study – Offspring Cohort		
	ALL (72 – 100)			ALL (79 – 103)			ALL (72 – 90)		
	LLFS (N = 1386)	CHS (N = 2964)		LLFS (N = 1369)	FHS – Original (N = 322)		LLFS (N = 575)	FHS – Offspring (N = 512)	
	Mean± SE	Mean± SE	Difference	Mean± SE	Mean± SE	Difference	Mean± SE	Mean± SE	Difference
Anthropometrics									
Height (cm)	160.3±0.24	160.8±0.31	0.5	160.0±0.23	159.7±0.43	−0.3	162.6±0.34	162.6±0.61	0.0
Weight (kg)	67.1±0.38	62.0±0.57	−5.1 ***	66.6±0.38	64.7±0.63	−1.9 *	71.3±0.59	69.0±1.06	−2.3
Body Mass Index (kg/m^2^)	26.0±0.14	23.9±0.20	−2.1 ***	25.5±0.14	25.4±0.28	−0.2	26.9±0.21	26.1±0.39	−0.8
Physical Function									
Gait Speed (m/s)	0.72±0.01	0.62±0.01	−0.1 ***	0.71±0.01	0.72±0.01	0.0			
Average Grip Strength (kg)	19.8±0.20	21.1±0.34	1.3 **	19.4±0.19	18.3±0.32	−1.1 **			
Cognitive Function									
Digit Symbol Substitution Test	27.0±0.37	22.0±0.58	−5.0 ***						
Mini-Mental State Exam	25.4±0.11	24.9±0.16	−0.5 **	25.2±0.12	24.4±0.29	−0.8 **	26.9±0.13	25.9±0.35	−1.0 **
Blood Pressure									
Systolic Blood Pressure^a^ (mmHg)	138.3±0.74	144.7±1.10	6.4 ***	138.6±0.75	140.0±1.50	1.4	138.2±0.99	141.5±1.94	3.3
Diastolic Blood Pressure^a^ (mmHg)	73.3±0.36	66.9±0.56	−6.4 ***	73.2±0.36	69.3±0.77	−3.9 ***	73.9±0.48	66.7±0.97	−7.2 ***
Pulse Pressure^a^	65.0±0.61	77.7± 0.91	12.6 ***	65.4±0.61	70.8±1.28	5.4 ***	64.3±0.79	74.7±1.66	10.4 ***
Fasting Glucose (mg/dL)^b^	96.1±0.65	109.0±1.17	12.9 ***				95.7±0.97	105.7±1.61	10.0 ***
Lipids									
Cholesterol (mg/dL)^c^	187.4±1.34	199.4± 1.94	12.1 ***	187.1±1.32	181.4±2.49	−5.7 *	189.2±1.88	189.1±3.14	−0.1
Low-Density Lipoprotein Cholesterol (mg/dL)	109.2±1.09	121.1± 1.77	11.9 ***	109.2±1.10	100.4±2.15	−8.8 ***	109.5±1.56	111.0±2.77	1.5
Kidney Function	109.6±1.79	129.5± 2.93	19.9 ***	110.2±1.80	139.3±4.49	29.1 ***	113.9±2.72	143.0±5.83	29.2 ***
Lung Function	1.20±0.01	1.26±0.02	0.1 **	1.21±0.01	1.33±0.03	0.12 ***	1.16±0.02	1.26±0.03	0.10 **
Peripheral Arterial Disease	2.24±0.02	2.33±0.03	0.1 *						
Ankle-Arm Index	1.06±0.01	0.95±0.01	−0.12 ***				1.13±0.01	1.08±0.02	−0.05 **

* p ≤ 0.05

** p ≤ 0.01

*** p ≤ 0.001

a Model additionally adjusted for anti-hypertensive medication use.

b Model additionally adjusted for oral hypoglycemic or insulin use.

c Model additionally adjusted for lipid-lowering medication use.

**Table 3b.** LLFS Offspring: Measurement means in LLFS and comparison cohorts adjusted for age, sex, race, education and smoking status.

	Cardiovascular Health Study			Framingham Heart Study – Original Cohort			Framingham Heart Study – Offspring Cohort			LONG LIFE Family Study – Controls		
	ALL (65 – 88)			ALL (79 – 88)			ALL (33 – 88)			ALL (31 – 88)		
	LLFS (N =776)	CHS (N=5804)		LLFS (N=51)	FHS – Original (N = 260)		LLFS (N=2321)	FHS – Offspring (N = 3247)		LLFS (N = 2744)	LLFS Controls (N = 783)	
	Mean± SE	Mean± SE	Difference	Mean± SE	Mean± SE	Difference	Mean± SE	Mean± SE	Difference	Mean± SE	Mean± SE	Difference
Anthropometrics												
Height (cm)	166.5±0.30	166.0±0.11	−0.4	162.1±1.01	162.2±0.62	0.0	167.2±0.22	166.3±0.14	−0.8 ***	167.3±0.21	167.7±0.24	0.5
Weight (kg)	77.5±0.58	73.0±0.23	−4.5 ***	77.2±2.25	70.5±1.16	−6.7 **	77.6±0.42	77.1±0.32	−0.5	77.6±0.39	77.4±0.52	−0.2
Body Mass Index (kg/m^2^)	27.9±0.21	26.4±0.08	−1.5 ***	29.3±0.82	26.3±0.43	−3.0 ***	27.7±0.15	27.8±0.12	0.1	27.7±0.14	27.4±0.18	−0.3
Physical Function												
Gait Speed (m/s)	1.11±0.01	0.92±0.00	−0.2 ***	0.92±0.03	0.90±0.02	0.0				1.12±0.01	1.16±0.01	0.0 ***
Average Grip Strength (kg)	29.2±0.28	29.7±0.13	0.5	22.8±1.04	21.4±0.56	−1.3				30.7±0.19	31.2±0.28	0.5
Cognitive Function												
Digit Symbol Substitution Test	44.4±0.52	42.5±0.19	−1.9 ***							48.3±0.34	47.4±0.45	−0.9 *
Mini-Mental StateExam	28.6±0.10	28.5±0.03	−0.1	27.6±0.69	27.8±0.26	0.2	28.7±0.06	28.5±0.05	−0.2 **	28.7±0.05	28.7±0.08	0.1
Blood Pressure												
Systolic Blood Pressure^a^ (mmHg)	134.1±0.84	133.5±0.37	−0.6	140.3±2.82	141.8±2.21	1.5	131.1±0.53	126.8±0.42	−4.3 ***	129.5±0.46	130.8±0.77	1.3
Diastolic Blood Pressure^a^ (mmHg)	77.9±0.42	70.5±0.20	−7.4 ***	74.0±1.13	71.3±1.11	−2.8	78.2±0.29	72.2±0.24	−5.9 ***	78.3±0.26	78.5±0.41	0.3
Pulse Pressure^a^	56.1±0.65	62.9±0.31	6.8 ***	66.2±2.39	70.6±1.86	4.4	53.0±0.37	54.6±0.36	1.6 ***	51.3±0.33	52.3±0.51	1.1
Fasting Glucose (mg/dL)^b^	79.8±0.79	108.2±0.45	28.3 ***				95.4±0.48	103.5±0.46	8.1 ***	94.6±0.46	97.2±0.80	2.6 ***
Lipids												
Cholesterol (mg/dL)^c^	203.5±1.87	212.4±0.76	8.9 ***	196.2±6.28	183.3±3.45	−12.9	205.8±1.17	202.6±0.84	−3.2 *	203.2±1.04	205.6±1.69	2.4
High-Density Lipoprotein Cholesterol (mg/dL)	61.1±0.77	53.9±0.29	−7.2 ***	61.1±2.33	55.0±1.53	−6.0 *	60.6±0.53	55.4±0.37	−5.2 ***	59.9±0.48	60.1±0.61	0.2
Low-Density Lipoprotein Cholesterol (mg/dL)	119.5±1.65	130.2±0.73	10.7 ***	112.6±5.78	100.9±3.00	−11.7	122.5±1.02	120.8±0.77	−1.7	120.6±0.91	123.7±1.51	3.1 *
Triglycerides (mg/dL)c	115.8±2.94	146.2±1.56	30.4 ***	112.9±8.20	137.0±6.74	24.1 *	115.2±2.09	132.3±1.84	17.1 ***	114.6±1.79	111.8±2.64	−2.8
Kidney Function												
Creatinine (mg/dL)d	1.01±0.01	1.03±0.01	0.0 *	1.10±0.04	1.14±0.04	0.04	1.00±0.01	1.09±0.01	0.09 ***	1.00±0.01	0.99±0.01	0.0
Lung Function												
Forced Vital Capacity (L)	3.13±0.03	3.14±0.01	0.0							3.35±0.02	3.37±0.03	0.0
Peripheral Arterial Disease												
Ankle-Arm Index	1.18±0.01	1.10±0.00	−0.08 ***				1.24±0.00	1.19±0.00	−0.05 ***	1.19±0.00	1.19±0.01	0.0

* p ≤ 0.05

** p ≤ 0.01

*** p ≤ 0.001

a Model additionally adjusted for anti-hypertensive medication use.

b Model additionally adjusted for oral hypoglycemic or insulin use.

c Model additionally adjusted for lipid-lowering medication use.

d Model additionally adjusted for height and weight.

The Long Life Family Study probands and offspring had significantly higher DBP and lower pulse pressures compared to CHS (Tables 3a and 3b). Pulse pressure was also significantly lower for LLFS proband generation compared with both FHS cohorts. Also, ankle-arm index was significantly better for both LLFS cohorts compared to those in the CHS and the FHS offspring cohort.

All lipid values were significantly better for LLFS proband and offspring generation compared to CHS, including lower total cholesterol, lower LDL-C and lower triglycerides. Triglycerides were lower for Long Life Family Study probands and offspring compared to both FHS cohorts. Both LLFS cohorts consistently exhibited higher levels of HDL-C, by about 5 mg/dL (Tables 3 and 3b) compared to CHS. Glucose levels were significantly lower by about 13 mg/dL for LLFS probands and 28 mg/dL for LLFS offspring compared to CHS. Kidney function was better for LLFS proband and offspring generations when compared to CHS, FHS original and FHS offspring as exhibited by lower creatinine levels.

**Figure 1.**
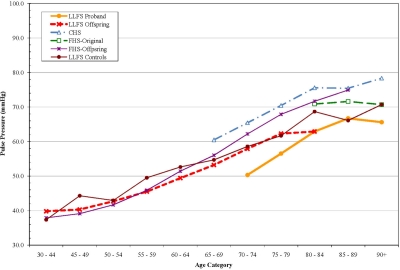
Pulse Pressure by age, adjusted for sex and anti-hypertensive medication use.

**Figure 2.**
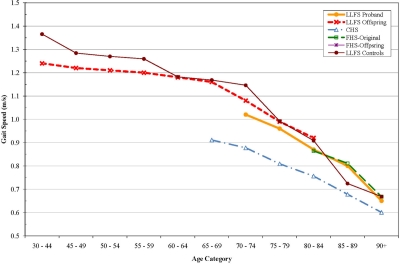
Gait Speed (m/s) by age, adjusted for sex.

**Figure 3.**
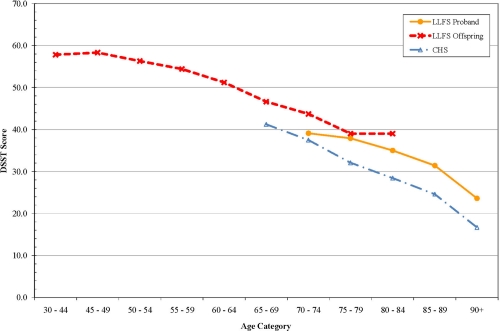
Digit Symbol Substitution Test (DSST) score by age.

For each measure, interactions with age were tested to determine if the differences between LLFS and the other cohorts might be greater at either extreme of age. There were no significant interactions however, age-specific analyses suggested several interesting patterns of age-related change. For pulse pressure, the differences between LLFS and other cohorts were most apparent after age 65. (Figure 1) Gait speed was higher in LLFS than the other cohorts (Figure 2). Digit symbol substitution test showed that LLFS participants had higher scores at every age, but with a similar age-specific decline (Figure 3).

## DISCUSSION

Probands and offspring of the LLFS cohort were less likely to have diabetes, chronic pulmonary disease and peripheral artery disease than the CHS and FHS cohort members. Measures of physical function and cardiovascular risk factors were more optimal in LLFS compared to the other groups. High density lipids were higher and triglycerides were lower in LLFS probands and offspring. This is consistent with previous reports of the children of centenarians having better lipid profiles than controls [20]. These findings suggest that the strategy of recruiting families with a history of longevity yielded rates of healthy aging not unlike that seen when recruiting offspring of centenarians [21].

We were also able to compare the oldest LLFS participants to participants in the NEC Study. These two groups appeared to be similarly exceptional in terms of history of major chronic disease although we did note a somewhat lower prevalence of cancer in the oldest members of LLFS. This is potentially due to the requirement in LLFS of an in-person examination, which excluded individuals with cancer under active treatment.

Comparisons between the offspring and their spouse controls showed trends consistent with better health in the LLFS offspring, but were not as strong as for some of the cross-cohort comparisons. These analyses were limited by the smaller sample size of this control group, but do suggest caution in attributing all differences to genetic factors in that part of the differences might be attributable to the shared environmental component of familial relationships.

One of the most consistent differences noted was a lower pulse pressure in the LLFS probands and offspring. The LLFS offspring had levels similar to individuals who were 5-10 years younger. This difference was due in part to a higher DBP in LLFS, as well as a lower systolic pressure. A low DBP with aging is associated with vascular stiffness as diastolic pressure tends to go down as SBP increases with age [22]. Thus, a widening pulse pressure has been viewed as a sign of accelerated vascular aging and will be an important phenotype for future genetic analysis of exceptional survival. Less widening of pulse pressure with age would be consistent with, consistent with a slower “rate of aging” phenotype.

Other age-specific differences suggested interesting patterns of age associated differences. The gait speed appeared to have a threshold of decline in LLFS with a much steeper decline after age 70, consistent with the idea that aging phenotypes can be manifest as a later onset of decline. Cognitive function as assessed by the DSST was higher at all ages in LLFS compared to CHS, consistent with the idea that they might have achieved a higher level of peak cognitive function earlier in life. This was not explained by their higher level of education. Conceptually, these patterns point to the need to consider “peak (or reserve) capacity [23], the “age of onset” of aging changes and the “rate of aging” as potentially distinct aspects of aging phenotypes that may vary across systems within an individual and require longitudinal data to establish. Longitudinal data in LLFS will be important to distinguish between these pathways to becoming successfully aged.

In spite of a lack of consistent difference in heart disease prevalence, the lower pulse pressure and other cardiovascular risk factors suggest that LLFS participants are healthier in terms of cardiovascular risk. Cardiovascular disease has a major environmental component in that there are several modifiable behavioral risk factors including diet, smoking and physical activity, and familial correlation of these factors could explain part of the associations seen. However, cardiovascular disease also has a significant genetic component. More detailed phenotyping with a continuous measure of atherosclerotic disease may improve our ability to detect familial associations and to sort out genetic vs. environmental components.

For the purpose of determining genetic contributions to longevity, “disability-free survival” as a phenotype may capture the joint effects of multiple chronic diseases of aging and the subject's ability to effectively deal with those diseases in order to maximize functional independence. Long Life Family Study participants had better physical functioning, based on assessments of gait speed and reported ADL difficulty. Physical functioning can be viewed as a summary measure of the impact of multiple conditions on health [24]. Gait speed differed between LLFS and other cohorts more than grip strength. This was unexpected given the robust ability of grip strength to predict mortality from middle age [24]. The grip strength protocol used in LLFS was slightly more difficult than used in the other cohorts and this may have obscured true differences. The Mini-Mental State Exam did not differ among these groups, but the measure used was designed to detect dementia and would be insensitive to detecting cognitive function differences within the normal range. More detailed analysis on a full battery of higher order cognitive function domains might reveal specific differences.

Functional status in older adults has been associated with various measures including muscle mass and strength, lung function, gait speed and weight loss [26-30]. Subclinical disease, especially cardiovascular disease, can be a strong determinant of function [31] as well as survival [32]. A challenge in the study of disability-free survival amongst aging adults predisposed to exceptional longevity is that its expression can occur late in very old age. In other words, disability per se is often not expressed until very late in life [33, 34]. Potentially, the continuous measures of function and the underlying physiology (e.g. gait speed, muscle strength, pulmonary function) may be more sensitive in detecting aspects of “rate of aging”, “age of onset” or “reserve capacity” and the predecessors of disability, which in turn could be valuable phenotypes for association and linkage studies of exceptional survival endophenotypes.

The Long Life Family Study is a novel cohort study. Future analysis will be strengthened by increasing the depth of the phenotypic characterization including longitudinal assessments. Several limitations of this analysis include the known protocol differences between studies and the potential that there are birth cohort differences that could not be examined. The differences found were independent of major behavioral risk factors such as smoking and education, but a major challenge to these assessments is the difficulty in fully capturing lifetime environmental risk factors, thus adjustment may not have been complete.

In summary, by selecting families characterized by having a strong history of longevity, we have identified individuals who have lower cardiovascular risk factors and higher levels of physical function. These findings suggest several priority phenotypes for inclusion in future genetic analysis to identify the loci contributing to exceptional survival.
